# *Cordyceps militaris* Fruit Body Extract Decreases Testosterone Catabolism and Testosterone-Stimulated Prostate Hypertrophy

**DOI:** 10.3390/nu13010050

**Published:** 2020-12-26

**Authors:** Kazuya Kusama, Mayuko Miyagawa, Koichiro Ota, Naoko Kuwabara, Kaori Saeki, Yuki Ohnishi, Yasuhiro Kumaki, Tomoyasu Aizawa, Toyokazu Nakasone, Shigemi Okamatsu, Hiroaki Miyaoka, Kazuhiro Tamura

**Affiliations:** 1 Department of Endocrine Pharmacology, Tokyo University of Pharmacy and Life Sciences, Tokyo 192-0392, Japan; kusamak@toyaku.ac.jp (K.K.); y154215@toyaku.ac.jp (M.M.); naokok@toyaku.ac.jp (N.K.); y154110@toyaku.ac.jp (K.S.); 2 Department of Biomolecular Organic Chemistry, Tokyo University of Pharmacy and Life Sciences, Tokyo 192-0392, Japan; otak@toyaku.ac.jp (K.O.); miyaokah@toyaku.ac.jp (H.M.); 3 Faculty of Advanced Life Science, Hokkaido University, Sapporo 060-8648, Japan; yonishi@sci.hokudai.ac.jp (Y.O.); kumaki@sci.hokudai.ac.jp (Y.K.); aizawa@sci.hokudai.ac.jp (T.A.); 4 Okinawa UKAMI Sericulture Co. Ltd. Heshiki, Nakijin-Son, Kunigami-Gun, Okinawa 905-0423, Japan; t-nakasone@okinawa-ukami.co.jp (T.N.); s-okamatsu@okinawa-ukami.co.jp (S.O.)

**Keywords:** *Cordyceps militaris*, testosterone, late-onset hypogonadism (LOH), prostate, benign prostate hyperplasia (BPH)

## Abstract

The androgens testosterone and dihydrotestosterone (DHT) are essential for a variety of systemic functions in mature males. Alteration of these hormones results in late-onset hypogonadism (LOH) and benign prostate hyperplasia (BPH). The fruit bodies of fungi of the genus *Cordyceps* have been regarded as folk medicine or health food with tonic and antifatigue effects. The extract from the fruit body of *Cordyceps militaris* parasitizing *Samia cynthia ricini* (CM) was evaluated as a novel-candidate natural product for ameliorating male andropause symptoms. To explore the effects of CM on LOH and BPH, CM was applied to rat models and cultured testicular cells and prostate cells. The concentrations of androgens in the serum and culture media were determined by ELISA. Expression of steroidogenic enzymes and androgen-related genes was evaluated by qPCR, and prostatic cell proliferation was assessed with the cell-viability assay. CM maintained the serum levels of testosterone and DHT, but inhibited testosterone-induced prostate hypertrophy. CM also increased the secretion of testosterone and DHT by primary testicular cells, with no changes in the mRNA expression of steroidogenic enzymes, but decreased the growth of prostatic cell lines. Our data suggest that CM could improve both LOH and BPH in males.

## 1. Introduction

Middle-aged and older males who present with symptoms including avolition, muscle pain, hot flashes, and loss of libido are regarded to have male menopausal disorders, and these symptoms are often accompanied by significant reductions in serum androgen levels [1]. Middle-aged and elderly males frequently exhibit the symptoms of late-onset hypogonadism (LOH), which is caused by androgen failure due to aging. The pathological decrease in androgen levels in LOH is caused by age-related dysfunction of testicular Leydig cells. LOH induces a variety of symptoms, including fatigue, depression, insomnia, and sarcopenia. Androgen-replacement therapy is one option for the treatment of LOH, but androgens have potent side effects and cannot be chronically used by most patients with andropause [1,2,3]. Meanwhile, benign prostate hyperplasia (BPH) is a well-known disease that impairs quality of life in aging males. Prostate hypertrophy causes urinary tract symptoms such as frequent urination and residual urine [4]. Various factors such as aging, oxidative stress, inflammation, and hormonal changes are involved in the pathogenesis of BPH [5,6], but the etiology is not fully understood. Antagonists of α-adrenoceptors and 5α-reductase inhibitors have been used for the management of BPH, because androgen dysregulation, particularly the excessive conversion of testosterone to dihydrotestosterone (DHT) by 5α-reductases, is thought to be involved in the pathogenesis of BPH [6]. Additionally, some plant-derived medicines that have anti-inflammatory and antiandrogenic effects, such as cernitin pollen, are also used in the treatment of BPH patients [7].

Androgens, including testosterone and DHT, are steroid hormones mainly produced by testicular Leydig cells in males [8]. Both androgens bind to androgen receptors and play roles in male sexual differentiation, adolescent development, and male fertility [9]. Testosterone is related to males’ willingness and motivation, and may also act protectively on impairments of the central nerve system such as depression and cognitive symptoms [2]. Testosterone synthesis progresses through several steps involving steroidogenic enzymes, including steroidogenic acute regulatory protein (StAR); cytochrome P450 family 17 subfamily A member 1 (CYP17a1); cytochrome P450 family 11 subfamily A member 1 (CYP11a1); and 3β-hydroxysteroid dehydrogenase (HSD3b1) [8]. Furthermore, testosterone is converted to DHT by 5α-reductases in the prostate, seminal vesicle glands, liver, and brain [10].

*Cordyceps militaris* belongs to the genus *Cordyceps*. Fungi of this genus grow as a parasite on the larvae of moths of the order Lepidoptera, and then form fruit bodies [11]. The dried fruit bodies of *Cordyceps* have been used for hundreds of years in traditional Asian medicine as a folk tonic agent [12,13] and are prized as a health food in Chinese cuisine. *Cordyceps militaris* has been described to have specific antifatigue activities without side effects [14,15]. In addition, *Cordyceps militaris* has been traditionally employed for the enhancement of sexual function, and recent reports suggest that the mushroom improves sperm quality and quantity [16], and testicular damage induced by bisphenol A [17]. In general, *Cordyceps militaris* contains cordycepin, ergosterol, and linoleic acid as the main bioactive compounds. However, the effects of components in *Cordyceps militaris* on the reproductive system have not yet been characterized using animal models. In the present study, we examined whether the extract of *Samia cynthia ricini*-derived *Cordyceps militaris* affects androgen metabolism and production using animal models of LOH and BPH, as well as *in vitro* cultured cells.

## 2. Materials and Methods

### 2.1. Preparation of the Extract from Cordyceps militaris Fruit Body (CM)

A microbial strain of *Cordyceps militaris* obtained from the National Institute of Technology and Evaluation (NBRC 100741, Chiba, Japan) was cultured in SDY medium. An efficient method for the growth of fruit bodies of *Cordyceps militaris* parasitizing *Samia cynthia ricini* (Ryukyu-kaso in Japanese) was recently established. The cultured media were filtered through gauze to remove the entangled hyphae. The liquid filtrate of hyphal bodies diluted with sterilized distilled water was injected into abdomens in the 3-day-old pupae of the *Samia cynthia ricini* as a host. The inoculated pupae were placed on a wet cotton cloth at 15 °C under the light to induce the development of the fruit bodies. After the primordium was formed, those pupae were placed in the dark at 18 °C under 90% humidity to enhance the production of fruit bodies. All harvested fruit bodies were immediately freeze-dried and stored at −20 °C. The freeze-dried fruit bodies of *Cordyceps militaris* were ground into a coarse powder and extracted two times with distilled water under reflux for 24 h. Solid materials were removed by centrifugation at 3800 rpm, and the resulting aqueous solution was freeze-dried to provide the dried extract. The powdered extract was dissolved in hot distilled water (100 mg/mL) and used for testing.

### 2.2. NMR Experiments and Analysis

NMR experiments and analysis were performed to identify and quantify chemical components in CM. A known amount (30–60 mg) of freeze-dried CM was suspended in phosphate buffer (50 mM Na_2_HPO_4_/NaH_2_PO_4_, pH 7.4, 10% *v*/*v* D_2_O) containing 1 mM TSP-d_4_ and 0.04% NaN_3_. These suspensions were centrifuged for 5 min at 15,000 rpm, and the supernatants were used for NMR measurement. All NMR data were obtained using a Bruker AVANCE III spectrometer (Bruker Biospin, Inc., Yokohama, Kanagawa, Japan) at 600 MHz with TXI z-gradient probe at 25 °C. The ^1^H NMR spectra were recorded using NOESY pulse sequence with presaturation for water suppression during a relaxation delay of 2.27 s and a mixing time of 0.1 s. The spectra were collected for each sample with 32,768 complex data points, sweep width of 7211.539 Hz, and 128 transients.

Acquired FIDs were zero-filled to 64 K, and an exponential line-broadening function of 0.2 Hz was applied before Fourier transformation. Processed spectra were phase- and baseline-corrected manually using Delta 5.0.4 (JEOL RESONANCE, Inc., Akishima, Tokyo) and referenced to TSP (δ0.00). For identification and quantification of chemical components, we performed targeted profiling with Chenomx NMR Suite 8.2 software package (Infocom, Corp., Tokyo, Japan), where ^1^H chemical shifts, peak multiplicity, and intensities were compared between experimental spectra and reference standards including amino acids and sugars. This database of reference standards does not include cordycepin, which is known as an important bioactive component of *Cordyceps sinensis;* therefore, we prepared standard NMR samples of cordycepin (FUJIFILM Wako Pure Chemical, Corp., Osaka, Japan), 10 mM dissolved in the same buffer, and measured NMR spectrum for identification of cordycepin in CM.

### 2.3. Animals and Tissue Preparation

Adult Wistar–Imamichi rats (8-week-old males; Institute for Animal Reproduction, Ibaraki, Japan) were maintained in a temperature- and light-controlled room (12 h light, 12 h dark cycle). All animal care and surgical procedures were approved by the Institutional Animal Care Committees (approval number: P19–42), in compliance with institutional guidelines for the care of experimental animals, which was in accordance with internationally accepted principles (the US guidelines/NIH publication). To explore the effect of CM on LOH, rats were castrated under isoflurane anesthesia. After 3 weeks, testosterone propionate (TP; 1 mg/kg, subcutaneous (s.c.), Fujifilm Wako Pure Chemical) and CM (20 mg/rat, oral (p.o.)) were administered to the castrated rats every other day or every day, respectively, for 12 days. Our preliminary data showed that this TP treatment maintained a normal range of serum testosterone concentrations (approximately 5–10 ng/mL). In the experiment to explore the effect of CM on BPH, high doses of TP (3 mg/kg, s.c.) and CM (20 mg/rat, p.o.) were administered daily to intact, noncastrated rats for 30 days. Animals were sacrificed the day after the final dose, and the serum, testes, prostate, and seminal vesicle glands were isolated. The collected tissues were frozen and stored in liquid nitrogen.

### 2.4. Measurement of Testosterone and DHT

The concentrations of testosterone and DHT in the serum and in culture media from rat-testicular cells were determined by ELISA (Dihydrotestosterone ELISA Kit, Abnova, Taipei, Taiwan; and Testosterone ELISA Kit, Cayman Chemical, Ann Arbor, MI, USA) according to the manufacturers’ instructions. The levels of testosterone and DHT in culture media samples were also assayed by liquid chromatography with tandem mass spectrometry (LC-MS/MS), which was carried out by ASKA Pharmaceutical Co. (Kanagawa, Japan).

### 2.5. RNA Extraction and Quantitative RT-PCR

Total RNA was extracted from prostate and testicular tissues or cultured testicular cells using ISOGEN reagent (Nippon Gene, Tokyo, Japan) according to the manufacturer’s protocol. Reverse transcription of isolated RNA was performed with the ReverTra Ace qPCR RT Kit (Toyobo, Osaka, Japan), and the generated cDNA was then subjected to qPCR amplification using PowerUP SYBR Green Master Mix (Thermo Fisher Scientific, Waltham, MA, USA). Primers are listed in Table 1. Calibration curves were used to confirm that the amplification efficiencies of each target gene and the reference genes (glyceraldehyde-3-phosphate dehydrogenase (*Gapdh*) and actin beta (*Actb*)) were comparable. Sequence Detection System software v2.3 (Thermo Fisher Scientific) was used to determine average threshold (Ct) values for each target [18].

### 2.6. Isolation of Primary Rat Testicular Cells

Rat testes were cut into small pieces, then washed thoroughly with Ca^2+^/Mg^2+^-free Hank’s balanced salt solution (HBSS; Fujifilm Wako Pure Chemical). Tissues were shaken in a digestion mixture of HBSS containing bovine serum albumin (1 mg/mL), type I collagenase (0.2 mg/mL, Sigma-Aldrich, St. Louis, MO, USA), type II collagenase (0.2 mg/mL, Sigma-Aldrich), type IV collagenase (0.2 mg/mL, Sigma-Aldrich), and DNase I (0.02 mg/mL, Nippon Gene) for 40 min at 37 °C. The enzyme-digested suspension was gently pipetted up and down in a 3 mL syringe (TERUMO, Tokyo, Japan) to disperse cell clumps, and then passed through a 70-µm nylon strainer to remove undigested tissue. Cells were resuspended in Dulbecco’s modified Eagle medium/F12 (DMEM/F12) (1:1) (Fujifilm Wako Pure Chemical) supplemented with serum and antibiotics as below.

### 2.7. Cell Culture and Evaluation of Androgen Secretion

Primary rat-testicular cells (5 × 10^4^ cells/well) were cultured on collagen type IA-coated dishes in DMEM/F12 medium (Fujifilm Wako Pure Chemical) supplemented with 15% horse serum, 2.5% fetal bovine serum (FBS), antibiotics, and antimycotics at 37 °C under 5 % CO_2_ in humidified air. The human prostate cell lines LNCaP and PC3 (Japanese Collection of Research Bioresouces Cell Bank, National Institutes of Biomedical Innovation, Health and Nutrition, Osaka, Japan) were cultured in RPMI 1640 medium (Nakalai Tesque, Kyoto, Japan) supplemented with 10% FBS, antibiotics, and antimycotics at 37 °C under 5% CO_2_ in humidified air. The human endometrial glandular epithelial cell line EM1 was also cultured in DMEM/F12 (Fujifilm Wako Pure Chemical) supplemented with 10% FBS, antibiotics, and antimycotics.

Rat-testicular cells were pretreated with CM (100 µg/mL) or cordycepin (0.5 mM, Fujifilm Wako Pure Chemical) for 1 h, and then treated with ovine LH (NIDDK-oLH-26; 10 or 100 ng/mL, provided by Dr. A. F. Parlow, National Hormone and Pituitary Program, Harbor-UCLA Medical Center, Torrance, CA, USA) or dibutyryl-cyclic AMP (Db; 0.1 or 0.5 mM, Tokyo Chemical Industry Co., Tokyo, Japan) for 1.5, 4, or 24 h to test the effects on steroidogenesis.

### 2.8. Cell Viability and Proliferation Assays

LNCaP, PC3, or endometrial EM1 cells (5 × 10^3^ cells) were seeded in 96-well culture plates and treated with TP (10 or 100 ng/mL, Fujifilm Wako Pure Chemical Corp.) in the presence or absence of CM (50, 100, 200, or 400 µg/mL) for 24 h. Cell viability and proliferation were assessed with the colorimetric WST-8 cell viability assay (Cell Counting Kit-8, Dojindo, Kumamoto, Japan) according to the manufacturer’s protocol [19].

### 2.9. Western Blotting

To test direct impact of CM on testosterone signaling, LNCaP cells were treated with TP (100 ng/mL) and dihydrotestosterone (10 ng/mL) with or without CM (100 µg/mL) for 24 h. Cells were lysed using RIPA buffer (Cell Signaling Technology, Tokyo, Japan), and the constituent proteins were separated by SDS-PAGE and transferred onto polyvinylidene difluoride membranes (Merck Millipore, Burlington, MA, USA). After blocking with Bullet Blocking One (Nacalai Tesque Inc., Kyoto, Japan), the membranes were incubated with mouse polyclonal antiphosphorylated androgen receptor (p-AR) antibody (1:500, Merck Millipore), or mouse monoclonal anti-GAPDH (1:5000, Fujifilm Wako Pure Chemical Corp.). Immunoreactive bands were detected using enhanced chemiluminescence (Merck Millipore) after incubation with horseradish peroxidase-labeled goat antimouse IgG (1:5000, Vector Laboratories, Burlingame, CA, USA).

### 2.10. Statistical Analysis

All experimental data from ELISA and qPCR analyses represent the results of three or more independent experiments. Data are expressed as the mean ± standard error of the mean (SEM). Significance was assessed using the Tukey-Kramer multiple comparisons test. A *p*-value < 0.05 was considered statistically significant.

## 3. Results

### 3.1. Quantification of the Amino Acids and Sugars in the Extract Components from Cordyceps militaris Fruit Bodies

**Figure 1:**
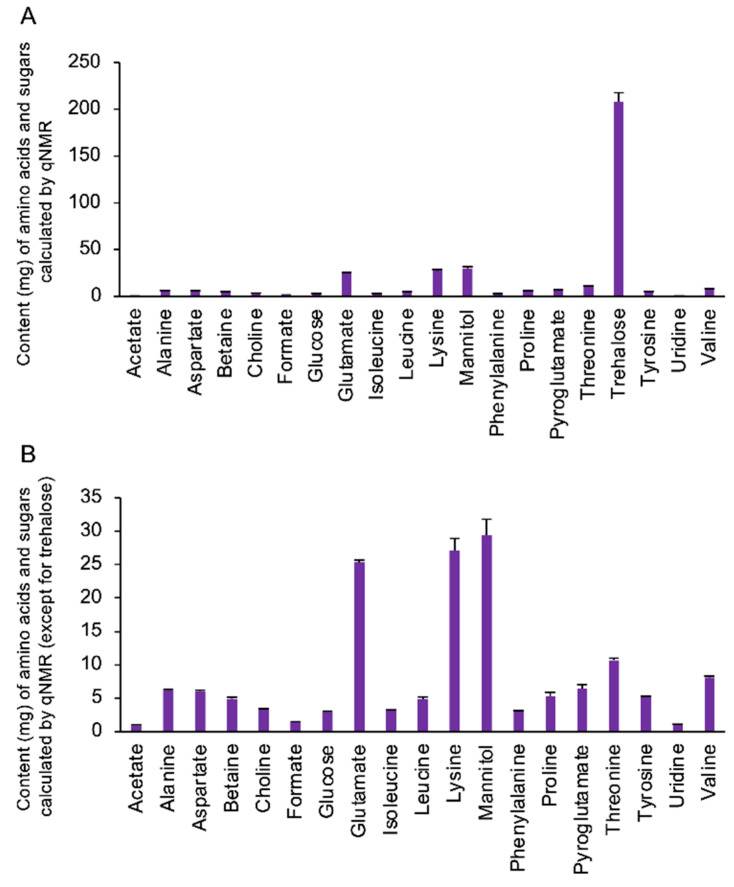
Summary of the identified components of the extract from *Cordyceps militaris* fruit bodies (CM). (**A**) The content of major components, derived by means of targeted profiling, is shown as the average content (mg) per gram (*n* = 4). (**B**) The average content of the components excluding the major component trehalose was shown.

Solution NMR was used as the preliminary analysis to identify the principal compounds in the extract of *Cordyceps militaris* from *Samia cynthia ricini.* Twenty major components of the extract were identified and quantified (Figure 1). Trehalose, which is well-known to contain fungi, algae, lichen, and crustacea, was identified as the most abundant component. While an extremely high concentration of trehalose (around 200 mg/g) was included, the concentrations of second groups containing a sugar alcohol mannitol, amino acids, glutamate, and lysine were several tens of mg/g. Cordycepin was also clearly identified (5.00 ± 0.10 mg) in this extract with spiking method.

### 3.2. The Effect of CM in a Rat Model of LOH

**Figure 2:**
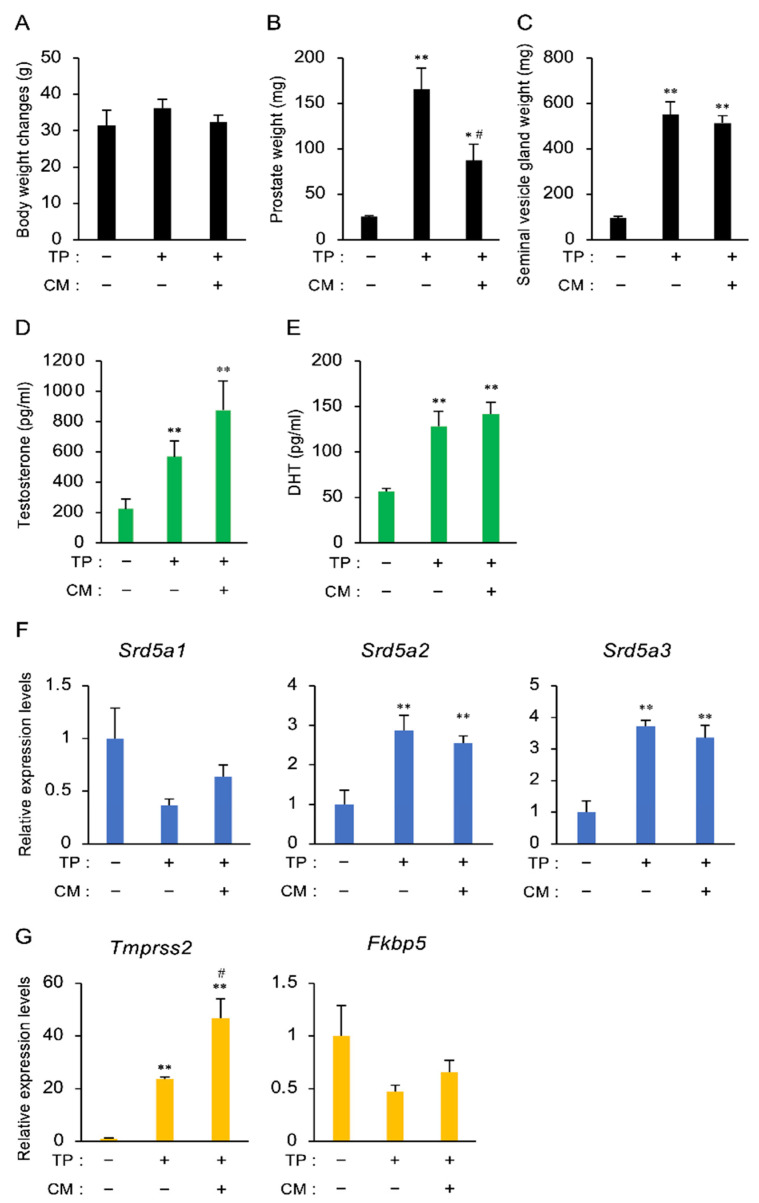
The effect of CM in a rat model of LOH. (**A**–**C**) The total body weight and the weights of the prostate and seminal vesicle glands on Day 12 after administration of TP (1 mg/kg, s.c.) and/or CM (20 mg/rat, p.o.) to castrated rats. Data are presented as the mean ± SEM of five rats. (**D**,**E**) Changes in the serum levels of testosterone and DHT. Both steroids were measured by ELISA. (**F**) RNA isolated from the prostate was subjected to qPCR to evaluate *Srd5a1*, *Srd5a2*, and *Srd5a3* mRNA expression. *Actb* and *Gapdh* were used as internal controls for RNA integrity. (**G**) Changes in *Tmprss2* and *Fkbp5* mRNA levels in the prostate. *Actb* and *Gapdh* were used as internal controls for RNA integrity. Data from five individual animals are shown. * *p* < 0.05 and ** *p* < 0.01 vs. intact, TP (-), and CM (-). ^#^ *p* < 0.05 vs. TP alone.

To investigate the effects of CM on LOH, TP and/or CM were administered to castrated rats for 12 days. There was no difference in body weight among the groups after 12 days (Figure 2A). As shown in Figure 2B,C, TP increased the weight of the prostate and seminal vesicle glands. TP-induced prostatic growth was inhibited by CM, while the increase in the size of the seminal vesicle glands was not affected. The serum levels of testosterone and DHT were increased by TP treatment (Figure 2D,E). Administration of CM tended to increase the levels of both hormones (Figure 2G).

To further examine whether CM affects testosterone-related gene expression in the prostate, the expression of steroid 5α-reductases (Srd5a1, Srd5a2, and Srd5a3) involved in the conversion of testosterone to DHT was evaluated by qPCR. The expression of Srd5a2 and Srd5a3 was increased by TP, but not influenced by CM (Figure 2F). In addition, changes in the expression of transmembrane protease, serine 2 (Tmprss2), and FK506-binding protein 5 (Fkbp5) [20,21], which are reported to be androgen-induced genes, were examined. CM administration enhanced Tmprss2 expression, but the expression of Fkbp5 was not changed in any group (Figure 2G). Thus, the inhibitory effect of CM on prostatic growth was observed in the rat exhibiting enhanced serum testosterone and prostatic Tmprss2 expression, suggesting that the reduction in prostate weight induced by CM may be independent of the serum level of testosterone.

### 3.3. The Effect of CM on BPH in Rats

**Figure 3:**
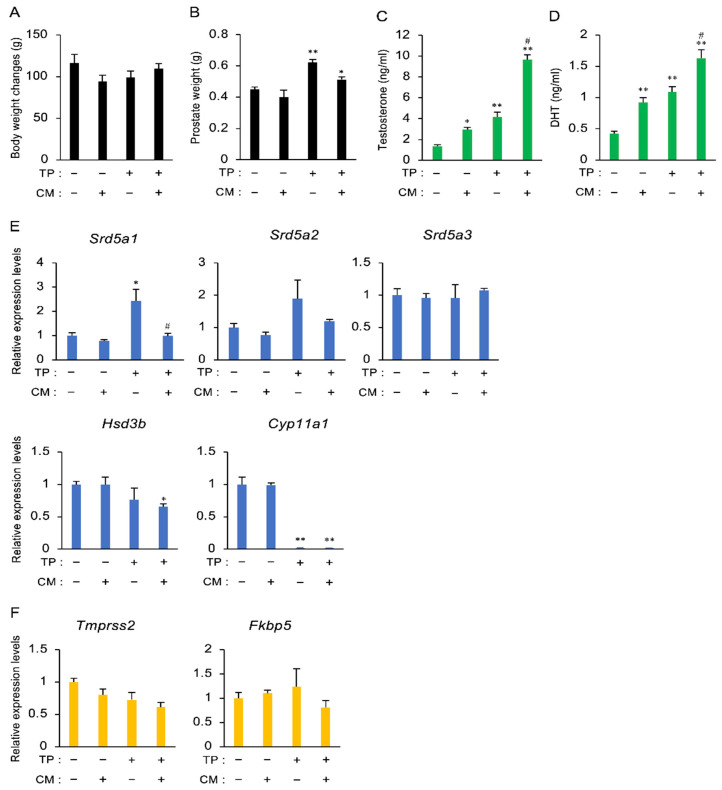
The effect of CM on BPH in rats. (**A**,**B**) Body and prostate weights on Day 30 after administration of TP (3 mg/kg, s.c.) and/or CM (20 mg/rat, p.o.). Data are presented as the mean ± SEM of five rats. (**C**,**D**) Changes in the serum levels of testosterone and DHT. Both steroids were measured by ELISA. (**E**) RNA isolated from the testis was subjected to qPCR analyses to evaluate the expression of *Srd5a1*, *Srd5a2*, *Srd5a3*, *Hsd3b*, and *Cyp11a1*. *Actb* and *Gapdh* were used as internal controls for RNA integrity. (**F**) Changes in *Tmprss2* and *Fkbp5* mRNA levels in the prostate. *Actb* and *Gapdh* were used as internal controls for RNA integrity. Data from five individual animals are shown. * *p* < 0.05 and ** *p* < 0.01 vs. intact, TP (-), and CM (-). ^#^ *p* < 0.05 vs. TP alone.

Based on the result that CM ameliorated the TP-induced increase in the weight of the prostate, we examined whether CM improved BPH using a model in which rats were injected with a high dose (3 mg/kg) of TP for 30 days. Although the body weight did not change (Figure 3A), the weight of the prostate was increased by TP, and this increase tended to be inhibited by CM (*p* = 0.06) (Figure 3B). Treatment with CM or TP alone increased the serum levels of testosterone and DHT, and combined treatment with CM and TP resulted in a greater increase than with either treatment alone (Figure 3C,D). The expression of Srd5a1 in the testis was elevated by TP, but TP-stimulated gene expression was suppressed by CM (Figure 3E). TP lowered Cyp11a1 expression, but only tended to decrease Hsd3b. No effects on mRNA levels of both steroidogenic enzymes testosterone-treated animals were observed. In addition, CM did not alter the expression of Tmprss2 or Fkbp5 in the prostate (Figure 3F).

### 3.4. The Effect of CM on the Proliferation and Androgen Receptor (AR) Activation of Prostate Cells

**Figure 4:**
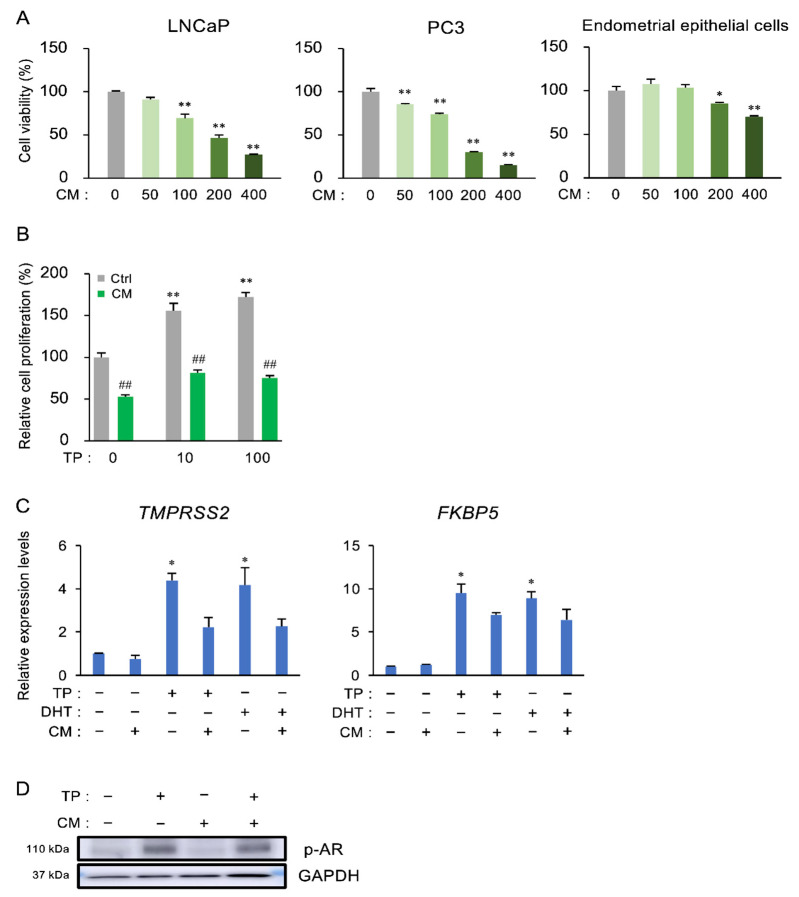
The inhibitory effect of CM on the proliferation of prostatic cells. (**A**) LNCaP cells, PC3 cells, and human endometrial glandular epithelial cell lines were treated with CM (50, 100, 200, or 400 µg/mL) for 48 h, and WST-8 assays were performed. ** *p* < 0.01 vs. CM (0). (**B**) LNCaP cells were pretreated with CM (100 µg/mL) for 1 h, then stimulated with TP (0, 10, or 100 ng/mL). WST-8 assays were performed after 24 h. ** *p* < 0.01 vs. Ctrl-TP (0). ^##^ *p* < 0.01 vs. Ctrl for each treatment. (C, D) LNCaP cells were stimulated with TP (100 ng/mL) or dihydrotestosterone (DHT, 10 ng/mL) in the presence of CM (100 µg/mL), and then qPCR analyses (**C**) for *TMPRSS2* and *FKBP5*, and western blotting for phosphorylated androgen receptor (**D**) were performed after 24 h. * *p* < 0.01 vs. intact.

We examined the effects of CM on cell proliferation in prostate cell lines (testosterone-sensitive LNCaP cells and castration-resistant PC3 cells), because CM inhibited the testosterone-stimulated hyperplasia of the prostate in models of both LOH and BPH. Unlike nonprostatic human endometrial epithelial cells, CM markedly decreased the viability of LNCaP and PC3 cells in a dose-dependent manner (Figure 4A). Furthermore, CM decreased TP-induced proliferation in LNCaP cells (Figure 4B). In addition, we observed that cordycepin inhibited both cell proliferation by about 70%, compared to control (data not shown).

To examine the influence of CM on androgen-induced genes and the receptor activation, LNCaP cells were stimulated with androgens (TP and DHT) in the presence of CM, and the expression of TMPRSS2 and FKBP5 was evaluated (Figure 4C). TP stimulated these expressions, but CM did not affect TP- or DHT-induced gene expression. In this condition, no changes were detected in the level of phosphorylated AR between the TP-treated group and the TP and CM-treated group (Figure 4D).

### 3.5. The Effect of CM on the Secretion of Androgens in Primary Rat Testicular Cells

**Figure 5:**
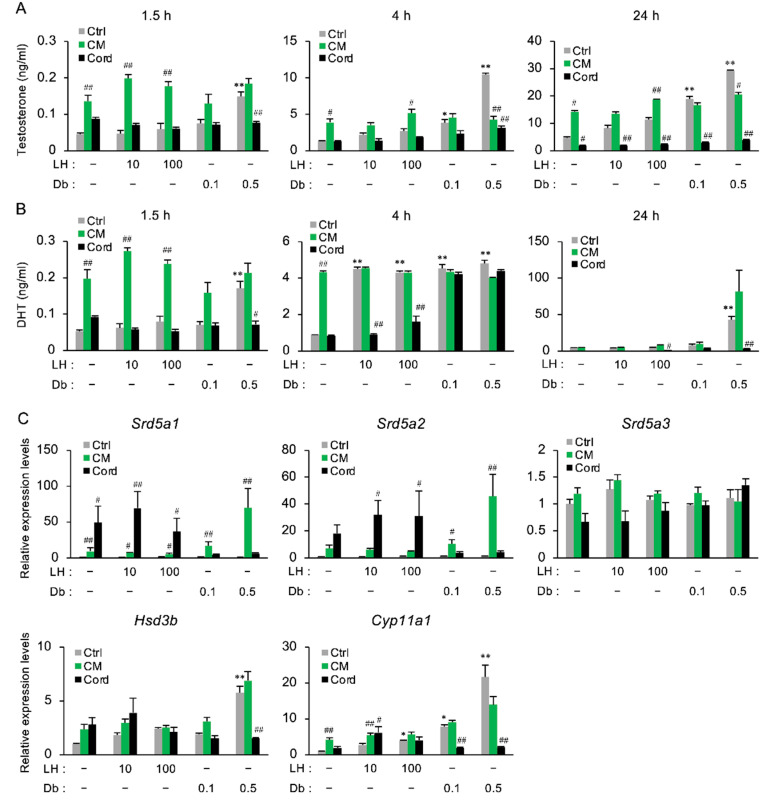
The effect of CM on the secretion of androgen in primary rat-testicular cells. Primary rat-testicular cells were treated with LH (10 or 100 ng/mL) or Db (0.1 or 0.5 mM) for 1.5, 4, or 24 h in the presence or absence of CM (100 µg/mL) or cordycepin (0.5 mM). (**A**,**B**) Changes in the levels of testosterone and DHT in the media. Both steroids were measured by ELISA. (**C**) RNA isolated from primary rat-testicular cells treated for 4 h was subjected to qPCR to evaluate *Srd5a1*, *Srd5a2*, *Srd5a3*, *Hsd3b*, and *Cyp11a1* mRNA expression. *Actb* and *Gapdh* were used as internal controls for RNA integrity. Data are from three independent *in vitro* culture experiments. * *p* < 0.05 and ** *p* < 0.01 vs. intact, TP (-), and CM (-). ^#^ *p* < 0.05 and ^##^ *p* < 0.01 vs. Ctrl for each treatment.

Testosterone production is regulated by luteinizing hormone (LH), which is released by the pituitary gland. LH induces the release of cyclic AMP (cAMP), which results in the activation of protein kinase A (PKA) and the expression of steroidogenic enzymes [8]. To further investigate the direct effect of CM on the secretion of testosterone and DHT from the testis, cultured primary rat-testicular cells were treated with LH or Db for 1.5, 4, or 24 h in the presence or absence of CM (Figure 5A,B). LH and Db increased the secretion of testosterone and DHT in a dose- and time-dependent manner, while CM increased the secretion of both hormones up to a threshold concentration independently of treatment with LH or Db (Figure 5A). The CM-induced increases in testosterone and DHT detected by ELISA at 1.5 h (in Figure 5A) were confirmed by LC-MS/MS (data not shown). Meanwhile, cordycepin treatment decreased the testosterone and DHT secretion. The expressions of *Srd5a1* and *Srd5a2* were increased by CM, but the expression of *Srd5a3* was not changed (Figure 5C, Supplementary Figure S1). Similar to the pattern of testosterone secretion, *Cyp11a1* was upregulated by CM up to a threshold value (Figure 5C, Supplementary Figure S1).

## 4. Discussion

We have demonstrated for the first time that an extract of the fruit body of *Cordyceps militaris* parasitizing the pupae of *Samia cynthia ricini* (CM) improved BPH in a rat model. CM increased both basal and TP-enhanced testosterone and DHT production. A similar trend was observed in an LOH model, although the CM-induced increase in serum levels of testosterone and DHT did not reach statistical significance in that model. CM did not alter the mRNA expression of key steroidogenic metabolizing enzymes in the testis and prostate except testicular Srd5a1 in a BPH model, indicating that CM did not alter the conversion of testosterone to DHT by increasing the expression of metabolic enzymes. In addition, CM increased the amount of testosterone and DHT in the culture media regardless of treatment with LH or Db in cultured testicular cells. However, in contrast to the above results in the in vivo BPH model, CM treatment of cultured testicular cells alone slightly, but significantly, upregulated the expression of *Srd5a1* and *Cyp11a1*. Furthermore, the pattern of steroidogenic enzyme gene expression did not parallel the changes in testosterone and DHT levels in cultured cells. These findings suggest that CM increases androgen levels by suppressing androgen catabolism and/or enhancing the androgen secretory process in *in vitro* testicular cells.

In addition to the positive action of CM on the maintenance of androgens in vivo and **in vitro**, CM inhibited TP-induced hypertrophy of the prostate in both LOH and BPH models. Under these conditions, TP stimulated the expression of prostatic *Tmprss2*, an androgen-regulated gene, and CM further increased its expression, suggesting that these changes may reflect the effects of CM-induced androgen production. Accordingly, these results imply that the CM-induced reduction in the weight of the prostate is independent of testosterone- and/or DHT-induced gene expression. Furthermore, CM decreased the viability of prostatic LNCaP and PC3 cells in a dose-dependent manner and decreased the testosterone-dependent proliferation of LNCaP cells. By contrast, CM had little effect on nonprostatic human endometrial epithelial cells. It is reported that *Cordyceps militaris* extract inhibited the proliferation of breast cancer, ovarian cancer, nonsmall-cell lung cancer, and colorectal cancer cells through androgen-independent signaling pathways [22,23,24,25]. Given that the CM-induced decrease in prostate hyperplasia was independent of the serum level of testosterone, CM may regulate the proliferation of prostate cells by inhibiting the activation of intracellular signal pathways other than those involved in testosterone signaling.

We have validated that a single treatment with a large dose of CM did not affect the body weight or the serum level of androgens (Supplementary Figure S2A–C). A single dose of CM (1 g/kg) also did not affect the expression of metabolic enzymes (Supplementary Figure S2D–I) or the serum level of alanine amino transferase (ALT) and had grossly normal liver, kidney and heart (data not shown). These results suggest the low potential of organ damage of CM. Cordycepin, one of the bioactive components of *Cordyceps militaris* or *Cordyceps sinensis*, has several bioactivities, including immunomodulatory effects, inhibition of tumor growth, and stimulation of adrenal hormones and testosterone secretion [11,26]. Although we have shown that cordycepin was a component of the extract used in this study, CM had the opposite effect of cordycepin on the secretion of androgens and the expression of metabolic enzymes in cultured testicular cells. It is reported that *Cordyceps sinensis* extract increased the growth of prostate cancer cells via the androgen receptor-dependent pathway [27], which differs from our results that CM reduced the weight of the prostate and the proliferation of prostate cancer cell lines independently of androgen levels. *Cordyceps militaris* contains various components in addition to cordycepin, including adenosine, polysaccharides including β-glucan, and ergosterol [13]. Unknown bioactive components or the interactive actions of multiple compounds in CM may exhibit various pharmacological effects. Intriguingly, it is reported that *C. militaris* from nature does not contain trehalose based on NMR analysis by other groups [28], but CM examined in this study contains an extremely high concentration. Although the direct relationship to the biological activity of major metabolites such as trehalose in CM is not clear at present, it is possible that the amounts of secondary metabolites having the direct biological activity also may be affected by the hosts and growing condition. Our next research step should focus on proper chemical characterization for identification of specific compound(s) that exhibit stimulatory action on testosterone levels and antiproliferative activity in prostate cells. Taken together, these findings indicate that CM, which contains several bioactive substances, including cordycepin, can improve LOH and BPH.

## 5. Conclusions

In conclusion, we indicate that the extract of *Cordyceps militaris* parasitizing *Samia cynthia ricini* maintained androgen levels and inhibited prostate hypertrophy, suggesting that CM, a novel natural product, may improve both LOH and BPH in aging males. Further studies, however, are required to characterize the components of CM that are effective for male menopause disorders and their mechanisms.

## Figures and Tables

**Table 1:** Primers for real-time PCR analyses.

Name (Accession No.)	Sequence	Product Length (bp)
Gapdh (NM_017008.4)	F: 5′- AAAGCTGTGGCGTGATGG -3′	96
	R: 5′- TTCAGCTCTGGGATGACCTT -3′	
Actb (NM_031144.3)	F: 5′- GGAGATTACTGCCCTGGCTCCTA -3′	150
	R: 5′- GACTCATCGTACTCCTGCTTGCTG -3′	
Srd5a1 (NM_017070.3)	F: 5′- GGTCTCCTCTCAAAACCTCAGG -3′	106
	R: 5′- GGAGGTCAAGTTCACAGCAAAC -3′	
Srd5a2 (NM_022711.4)	F: 5′- TATACTCCTTTCTGCCCAGGGA -3′	171
	R: 5′- GTTTTGACTGCAGAACTTCCCC -3′	
Srd5a3 (NM_001013990.1)	F: 5′- CTTTGGGCTTTGCTCAGAACTC -3′	137
	R: 5′- AGACTATGGACCCAGAGGAACA -3′	
Hsd3b (NM_001007719.3)	F: 5′- CCAGTGTGCCAGCCTTCATCTAC -3′	104
	R: 5′- GCTTTCATGATGCTCTTCCTCATG -3′	
Cyp11a1 (NM_017286.3)	F: 5′- AGTTCAGATGCCTGGAGGAAAG -3′	165
	R: 5′- GTCCCCTGAGAACTTTCCAGAG -3′	
Tmprss2 (NM_130424.3)	F: 5′- ACAACTCAAGCCTCAACACC -3′	150
	R: 5′- CTTCCAAAGCAAGCCAGCAG -3′	
Fkbp5 (NM_001012174.1)	F: 5′- TTCCAGTCGTGACAGAAACG -3′	98
	R: 5′- CGCCTTTCTTCATGGTAGACAC -3′	
TMPRSS (NM_001135099.1)	F: 5′- CCTCTAACTGGTGTGATGGCGT -3′	121
	R: 5′- TGCCAGGACTTCCTCTGAGATG -3′	
FKBP5 (NM_004117.4)	F: 5′- CTGCAGAGATGTGGCATTCACT-3′	75
	R: 5′- TCCAGAGCTTTGTCAATTCCAA -3′	

F: Forward, R: Reverse.

## Supplementary Materials

The following are available online at https://www.mdpi.com/2072-6643/13/1/50/s1, Figure S1. Figure S2.

## Abbreviations

CM	*Cordyceps militaris* parasitizing *Samia cynthia ricini*
LOH	Late-onset hypogonadism
BPH	Benign prostate hyperplasia
DHT	Dihydrotestosterone,
DMEM/F12	Dulbecco’s modified eagle’s medium/F12
Db	Dibutyryl-cyclic AMP
PBS	Phosphate-buffered saline
