# Supplementary figures and images for: Cordyceps militaris Fruit Body Extract Decreases Testosterone Catabolism and Testosterone-Stimulated Prostate Hypertrophy
Source: Nutrients. 2020 Dec 26;13(1):50. doi: 10.3390/nu13010050 (PMC7824671; doi:10.3390/nu13010050)

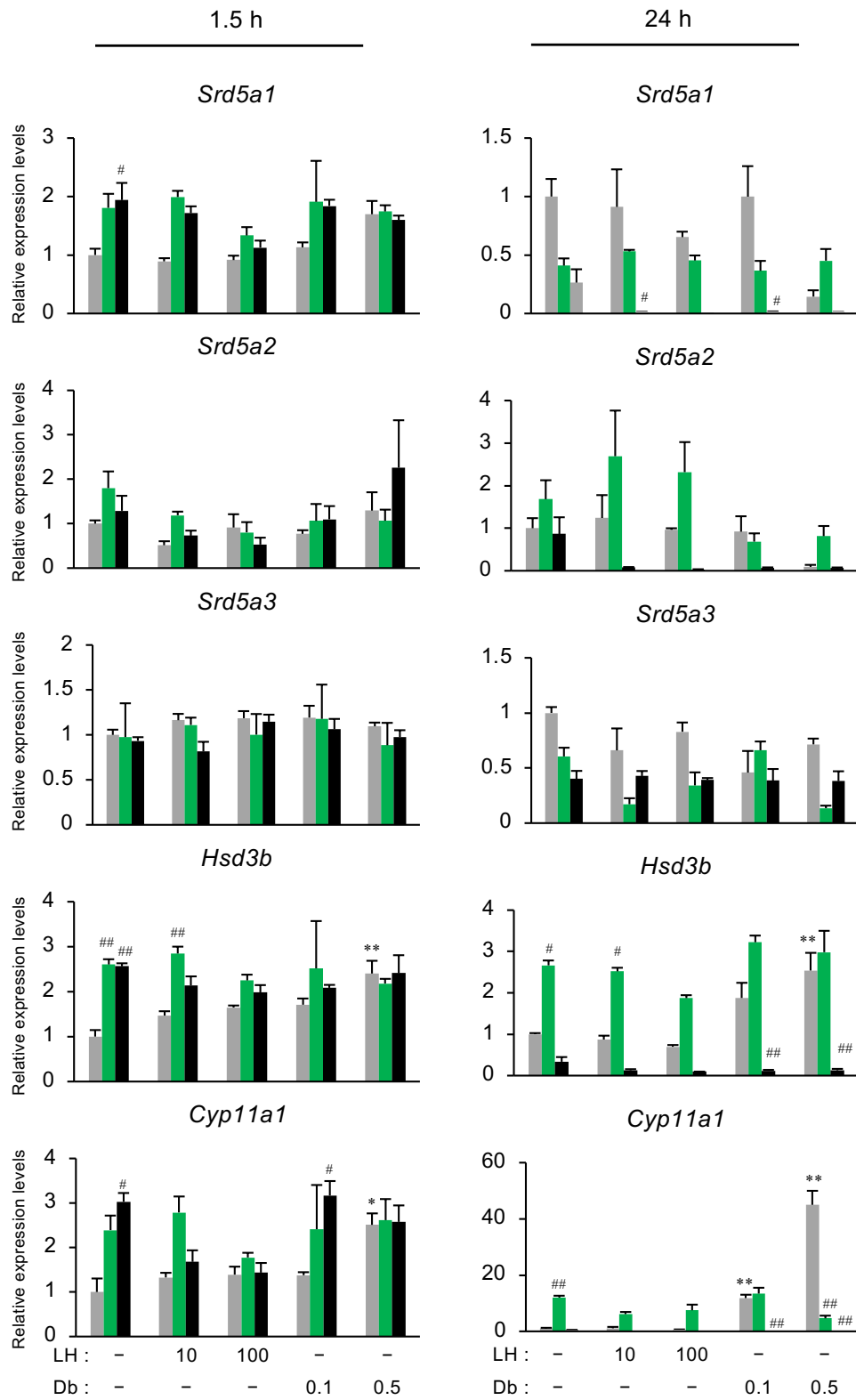

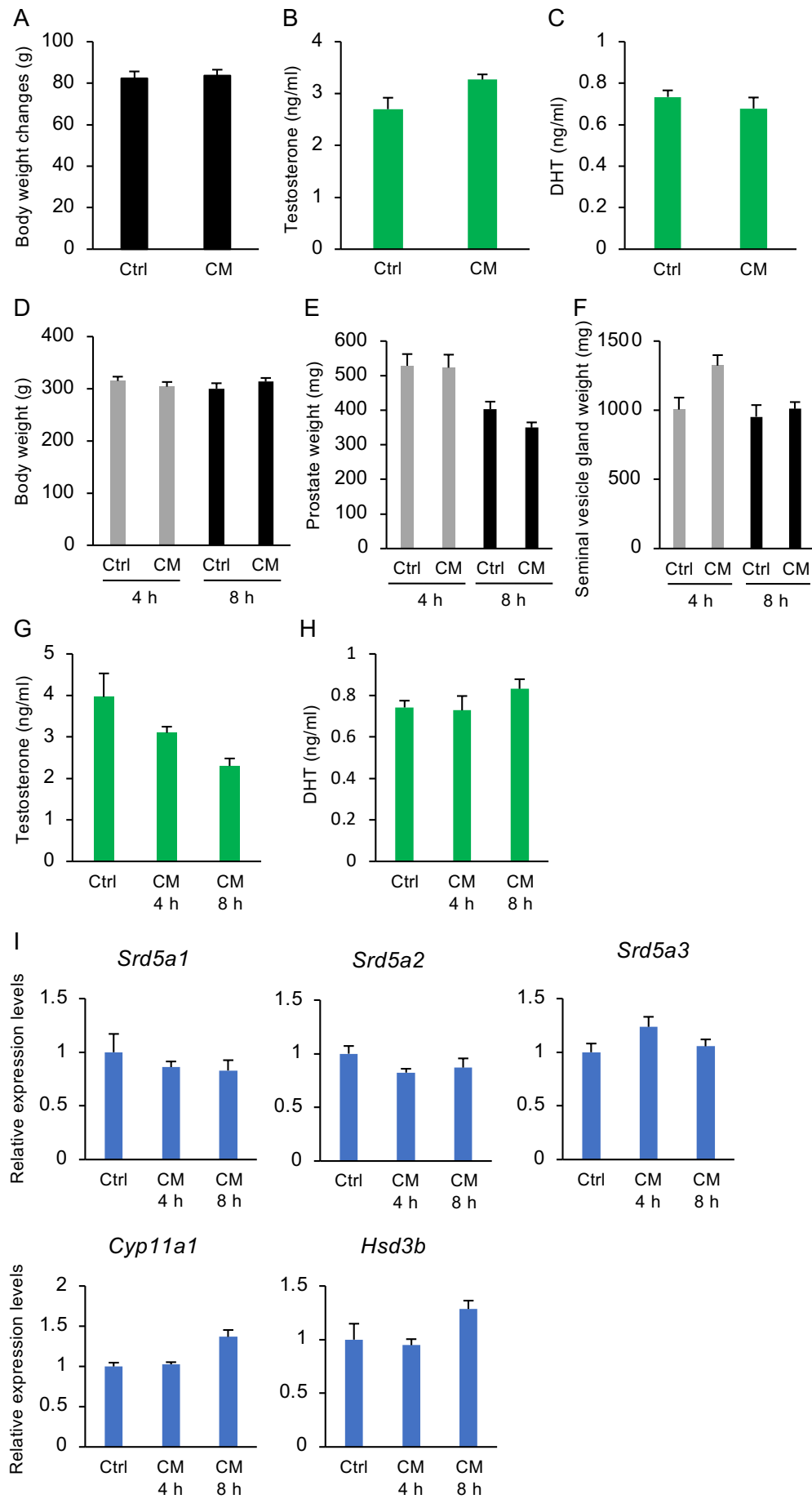

Supplement: Supplementary file 1 [file nutrients-13-00050-s001.pdf]
